# Retraction: A highest stable cluster Au_58_ (*C*_1_) re-optimized *via* a density-functional tight-binding (DFTB) approach

**DOI:** 10.1039/c8ra90100g

**Published:** 2018-12-10

**Authors:** Andrew Shore

**Affiliations:** Royal Society of Chemistry Thomas Graham House, Science Park, Milton Road Cambridge CB4 0WF UK advances-rsc@rsc.org

## Abstract

Retraction of ‘A highest stable cluster Au_58_ (*C*_1_) re-optimized *via* a density-functional tight-binding (DFTB) approach’ by K. Vishwanathan *et al.*, *RSC Adv.*, 2018, **8**, 11357–11366.

(1) M. Springborg wishes to resign as co-author to the above article. Professor Springborg has declared that he was unaware of this submission, did not approve the manuscript and disagrees with some of its scientific conclusions.

The corrected authorship list and affiliations for this paper are as follows:

K. Vishwanathan*^a^

^a^ Physical and Theoretical Chemistry, University of Saarland, 66123 Saarbrücken, Germany. E-mail: vishwa_nathan_7@yahoo.com; Tel: +49-0151-63119680

(2) The Royal Society of Chemistry hereby wholly retracts this *RSC Advances* article due to unattributed text and equation overlap with a diploma thesis completed by Ingolf Warnke, under the supervision of Professor M. Springborg, at the University of Saarland in 2007 (ref. 1). There are also portions of text overlap with papers published by different authors cited as ref. 36, 38, 39, 43 and 44 in the paper. In addition, there is considerable unattributed text overlap with the author’s own previously published papers cited as ref. 52–55 in the article.

Signed: Andrew Shore, Executive Editor, *RSC Advances*

Date: 4^th^ December 2018

## Supplementary Material
